# What We Know and Would Like to Know about CDKL5 and Its Involvement in Epileptic Encephalopathy

**DOI:** 10.1155/2012/728267

**Published:** 2012-06-17

**Authors:** Charlotte Kilstrup-Nielsen, Laura Rusconi, Paolo La Montanara, Dalila Ciceri, Anna Bergo, Francesco Bedogni, Nicoletta Landsberger

**Affiliations:** ^1^Theoretical and Applied Sciences, Division of Biomedical Research, University of Insubria, 21052 Busto Arsizio, Italy; ^2^San Raffaele Rett Research Center, Division of Neuroscience, San Raffaele Scientific Institute, 20132 Milan, Italy

## Abstract

In the last few years, the X-linked serine/threonine kinase cyclin-dependent kinase-like 5 (CDKL5) has been associated with early-onset epileptic encephalopathies characterized by the manifestation of intractable epilepsy within the first weeks of life, severe developmental delay, profound hypotonia, and often the presence of some Rett-syndrome-like features. The association of CDKL5 with neurodevelopmental disorders and its high expression levels in the maturing brain underscore the importance of this kinase for proper brain development. However, our present knowledge of CDKL5 functions is still rather limited. The picture that emerges from the molecular and cellular studies suggests that CDKL5 functions are important for regulating both neuronal morphology through cytoplasmic signaling pathways and activity-dependent gene expression in the nuclear compartment. This paper surveys the current state of CDKL5 research with emphasis on the clinical symptoms associated with mutations in *CDKL5*, the different mechanisms regulating its functions, and the connected molecular pathways. Finally, based on the available data we speculate that CDKL5 might play a role in neuronal plasticity and we adduce and discuss some possible arguments supporting this hypothesis.

## 1. Introduction

The kinase CDKL5 (Cyclin-Dependent Kinase-Like 5) was initially identified through a positional cloning study aimed at isolating disease genes mapping to Xp22. Sequence analysis revealed homologies to several serine-threonine kinase genes and identified one protein signature specific for this subgroup of kinases, therefore, leading the authors to name the gene *STK9* (Serine Threonine Kinase 9) [1]. Within the next five years, mutations in this gene were found in epileptic patients; in 2003, Vera Kalscheuer suggested STK9 to be a chromosomal locus associated with X-linked infantile spasms (ISSX) [2]. In particular, by sequence comparison, the authors discussed the resemblance of the kinase domain of STK9 to the MAP kinase family and hypothesized a role in the MAP kinase cascade, a hypothesis still to be tested. Given the strong similarity to some cell division protein kinases [2], the *STK9 *gene subsequently appeared as *CDKL5/STK9* and, eventually, got renamed *CDKL5*.

A study of the evolutionary conservation of the human CDKL5 protein sequence (GeneBank accession n°. CAI42485) identified several orthologs in vertebrates with large blocks of homology diffused through the whole protein. Fish orthologs diverge the most from the human protein [3], while no orthologs have been found in invertebrates or in plants (unpublished data). 

Despite the clear involvement of CDKL5 for proper brain development, as evidenced from the available clinical and molecular data, CDKL5 functions are still far from being understood. This paper examines the current status of CDKL5 research, highlighting the clinical symptoms involving CDKL5, the diverse isoforms so far described and their regulation of expression, the molecular pathways associated with it, and the most urgent studies required to better understand its functions and its possible involvement in neuronal plasticity.

## 2. Clinical Aspects Associated with *CDKL5* Mutations

Even though the first patients mutated in *CDKL5 *were two girls affected by X-linked infantile spasms [2], subsequent cases were reported in female patients with a clinical phenotype mimicking Rett syndrome (RTT), which is in most cases caused by mutations in *MECP2* [4, 5]. Because of that, the first genetic screenings for *CDKL5 *mutations occurred mainly in cohorts of patients with RTT syndrome, or a variant of it, with no *MECP2 *mutations. By comparing the clinical phenotypes of patients with *CDKL5 *mutations already described in literature, it became clear that almost all of them presented early epilepsy, starting from 10 days to 3 months after birth. Therefore, in the last years, *CDKL5 *screenings have been extended to cohorts of both genders in which patients were characterized by epileptic encephalopathy. Interestingly, Intusoma et al. [6] suggested in a recent paper that screening among patients having intractable seizures with an onset before 6 months of age gives a higher score than screening among *MECP2*-negative RTT patients; this score is even increased when RTT-like features are shown as well. In detail, the authors estimated that clinical sensitivity among females with intractable seizures starting before the ages of 12, 6, and 3 months were 4.7, 11.6, and 14.3%, respectively. Stunningly, this value rises to 31.3% when some typical RTT symptoms are included in the phenotype [6]. Importantly, these results are in good concordance with other publications.

Albeit few milder cases have been reported, the clinical phenotype of girls mutated in *CDKL5 *is in general comparable between several reports [7–9]. In particular, early drug-resistant epilepsy, usually starting in the first months of life, tends to be the most common feature. Seizures are in general highly polymorphic and many different seizure types can also occur in the same patient, changing with time. Complex partial seizures, infantile spasms, myoclonic, generalized tonic-clonic, and tonic seizures have all been reported. Very often, patients treated with antiepileptic drugs face a seizure-free honey-moon period, which, unfortunately, is followed by relapses. The clinical and EEG data, available so far, do not permit to recognize any exact pattern; conversely, no specific abnormalities have been clearly detected by MRI [2, 10–12]. In addition, it has been proposed that stereotypic hand movements, severe hypotonia, and impaired psychomotor development are usually associated with *CDKL5 *mutations and common to the general clinical manifestation of RTT patients [7–9, 12]. Whereas most of the RTT features presented by *CDKL5 *patients become evident when they get older [12], these patients lack the apparent normal development followed by regression that is usually considered a diagnostic criteria for Rett syndrome [13]. Furthermore, differently from RTT, *CDKL5 *patients are generally characterized by residual hand use, poor eye fixation with avoidance of eye contact, visual impairment, and feeding difficulties. Indeed, cortical visual loss has been described by several reports [12, 14–16], and Moseley and colleagues have recently reported that many young CDKL5-positive children exhibit significant dysphagia, requiring PEG/PEJ placement [16]. Eventually, autonomic features such as breathing irregularities, cold extremities, and gastrointestinal disturbances might occasionally be found in *CDKL5 *patients, whereas they are consistently found in Rett syndrome.

An interesting diversity between patients mutated in *MECP2* or *CDKL5* emerges from the clinical severity shown by male patients with *CDKL5 *mutations with respect to those of the same gender carrying *MECP2 *mutations. Indeed, it is generally accepted that *MECP2 *mutations causing classic Rett syndrome in females lead to encephalopathy and death in males with a normal chromosome complement within the first years of life. The same mutations, instead, lead to Rett syndrome in XXY backgrounds or in males with somatic mosaicisms. Eventually, mutations with minor clinical relevance in females can lead to RTT in males [17]. On the contrary, the most recent reports suggest that clinical severity does not differ between males and females with *CDKL5 *mutations [10, 18, 19]. Furthermore, whereas *MECP2 *mutations are rarely found in males, Liang et al. estimated that, in a cohort of patients affected by epileptic encephalopathy, *CDKL5 *mutations have a frequency of 5% in males and 14% in females [10]. While these studies suggest that *CDKL5 *testing should be considered independently of the gender, they also lead to an important question: why do mutations in an X-linked gene lead to a similar outcome in females and males? Even though no answers are yet available, we believe that, in the future, it will be important to address whether CDKL5-depleted neurons affect the phenotype of wild-type neurons. Anyhow, these results suggest that whereas a major source of the phenotypic variability associated with *MECP2 *mutations is given by the pattern of X chromosome inactivation, this does not seem to influence the clinical outcome determined by CDKL5. Accordingly, a study describing a male with Klinefelter syndrome (47, XXY) and a large CDKL5 COOH-truncation presenting a phenotype comparable to those of other CDKL5-positive boys suggests that the presence of a wild-type *CDKL5 *allele and a balanced pattern of X-inactivation does not reduce the severity of the disease [20].

## 3. The *CDKL5* Gene and Protein Isoforms

**Figure 1:**
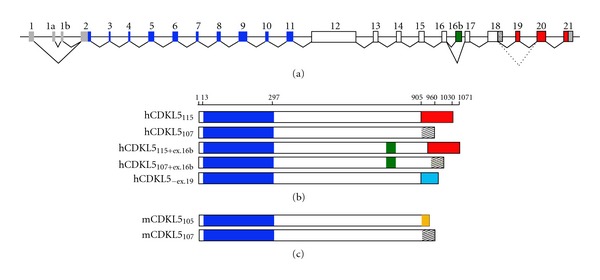
The genomic structure of *CDKL5* and its splice variants. (a) The human *CDKL5* gene with nontranslated sequences in grey and exons encoding the catalytic domain in blue. Exons encoding the common C-terminal region appear in white, whereas isoform-specific sequences are shown in red, green, and as hatched. (b) hCDKL5 protein isoforms differing in the C-terminal region. CDKL5_115_ [22] contains the primate-specific exons 19–21. In CDKL5_107_ [3], intron 18 is retained. The inclusion of exon 16b would generate CDKL5_115+ex.16b_ and/or CDKL5_107+ex.16b_ [21, 23]. hCDKL5_−ex19_ is a hypothetical splice variant in which exon 19 is excluded generating an alternative C-terminus (light blue; personal communication Limprasert.) (c) The murine CDKL5 isoforms. mCDKL5_105_ harbors a distinct C-terminal region encoded by a mouse-specific exon 19 (orange). As in humans, the retention of intron 18 generates the common CDKL5_107_ isoform [3].

The human *CDKL5* gene occupies approximately 240 kb of the Xp22 region and is composed of 24 exons of which the first three (exons 1, 1a, 1b) are untranslated, whereas the coding sequences are contained within exons 2-21 (Figure 1). Two splice variants with distinct 5′UTRs have been found: isoform I, containing exon 1, is transcribed in a wide range of tissues, whereas the expression of isoform II, including exons 1a and 1b, is limited to testis and fetal brain [2, 3]. Alternative splicing events lead to at least three distinct human protein isoforms. The original *CDKL5* transcript generates a protein of 1030 amino acids (CDKL5_115_; 115 kDa). While CDKL5_115_ is expressed mainly in testis, two recently identified transcripts are likely to be relevant for CDKL5 brain functions [3, 21]. As depicted in Figure 1, these two isoforms are characterized by an altered C-terminal region. Importantly, one of these (CDKL5_107_ in Figure 1) is common in a number of species, including mouse, which renders *Cdkl5* mouse models of significant relevance for studying CDKL5 functions [3]. Interestingly, an alternative splice variant, containing yet another distinct C-terminus, has been predicted through a bioinformatics simulation (ECgene analysis; personal communication Dr. Limprasert).

Interestingly, two isoforms selectively expressed in rat neurons and glial cells have recently been described [24]. By western blotting of mouse CDKL5 in a pure glial culture, a weak signal sensitive to siRNA of CDKL5 and migrating below the major band in a brain extract can be detected (our unpublished results). This suggests that a glial-specific CDKL5 isoform is expressed also in mouse.

## 4. *CDKL5* Mutations and Their Influence on the Phenotypic Outcome

**Figure 2:**
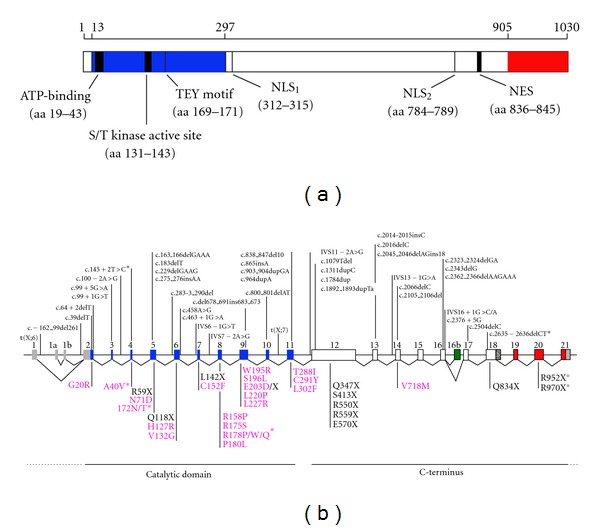
Pathogenic *CDKL5* mutations. (a) Schematic representation of CDKL5_115_ with the functional domains and signatures indicated. *NLS: *nuclear localization signal; *NES: *nuclear export signal. (b) All mutations in *CDKL5* reported to date are indicated corresponding to their location within the gene. Mutations shown above the *CDKL5* gene are deletion and frame shift mutations as well as splice variants indicated with cDNA nomenclature. Missense and nonsense mutations (fuchsia and black, resp.) are represented with amino acid nomenclature below the *CDKL5* gene. *: recurrent mutations; °: uncertain pathogenicity. The indicated mutations have been referred by: t(X;6) [2]; c.-162_99del261 [25]; c.39delT [10]; c.64+2delT [12]; c.99+1G>T [12, 26]; c.99+5G>A [8, 27]; c.100-2°>G [9]; c.145+2T>C [12, 25, 28]; c.229delGAAG [12]; c.183delT [5]; c.163_del166delGAAA [18, 29]; c.275_276insAA [8]; c.283-3_290del [8]; c.del458A>G [9]; c.463+1G>A [9]; IVS6-1G>T [30]; IVS7-2A>G [31]; c.del678_691ins683_673 [30]; c.800_801delAT [12, 26]; t(X;7) [2]; c 838_847del10 [22]; c.865insA [12]; c.903_904dupGA [25]; c.964dupA [18]; IVS11-2°>G [30]; c.1079Tdel [10]; c.1311dupC [12, 26]; c.1784dup [8]; c.1892_1893dupTA [12, 26]; c.2014-2015insC [10]; c.2016delC [12]; c.2045_2046delAGins18 [12, 26]; IVS13-1G>A [5]; c.2066delC [18]; c.2105_2106del [8]; c.2323_2324delGA [12, 26]; c2343delG [22]; c.2362-2366delAAGAAA [30]; IVS16+1G>C/A [30, 31]; c.2376+5G [25]; c.2635_2636delCT [12, 26, 29]; G20R [32]; A40V [9, 12, 26, 33]; R59X [30, 34]; N71D [7]; I72N [31]; I72T [25]; Q118X [12, 26]; H127R [25]; V132G [7]; L142X [12]; C152F [4]; R158 [35]; R175S [4]; R178P [9, 19]; R178W [7, 9]; R178Q [10]; P180L [30]; S196L [32]; E203D [7]; E203X [36]; L220P [12, 26, 33]; L227R [9]; T288I [19]; C291Y [19]; L302F [10]; Q347X [7, 10]; S413X [10]; R550X [25, 28]; R559X [10, 20]; E570X [36]; V718M [12]; Q834X [12, 26, 37]; R952X [6]; R970X [38].

The CDKL5 protein belongs to the CMGC family of serine/threonine kinases (including cyclin-dependent kinases (CDKs), mitogen-activated protein kinases (MAP kinases), glycogen synthase kinases (GSK), and CDK-like kinases) and is characterized by an N-terminal catalytic domain (amino acids 13–297), homologous to that of the other CDKL-family members. CDKL5 is unique in this family of kinases as it displays an unusual long tail of more than 600 amino acids without obvious similarity to other protein domains but with a high degree of conservation between different CDKL5 orthologs that differ only in the most extreme C-terminus. Besides the ATP-binding region and the serine/threonine protein kinase active site (amino acids 13–43 and 131–143, resp.), CDKL5 is characterized by a Thr-Xaa-Tyr motif (TEY) at amino acids 169–171, whose dual phosphorylation is normally involved in activating, among others, kinases of the MAP kinase family. Moreover, putative signals for nuclear import (NLS) and export (NES) have been found in the C-terminus of the protein, as shown in Figure 2.

The limited number of CDKL5 targets has not yet allowed drawing a consensus sequence for this kinase; however, the presence of a critical arginine-residue in the kinase subdomain VIII suggests that CDKL5 might be a proline-directed kinase. Moreover, as some of the other CMGC protein kinases, CDKL5 appears capable of auto-phosphorylating its TEY motif [39].

Almost 90 different *CDKL5 *patients, harboring a wide range of pathogenic mutations, have been described so far, including missense and nonsense mutations, small and large deletions, and frameshifts and aberrant splicing (Figure 2). Even though the small number of cases does not permit to draw any conclusive data, hot-spots have been suggested so far only for few mutations (indicated with an asterisk in Figure 2). By analyzing the distribution of missense mutations, it appears evident that they localize mainly in the catalytic domain, thus confirming the relevance of the kinase activity of CDKL5 for proper brain function and/or development. On the contrary, truncating mutations can be located anywhere in the gene, leading to CDKL5 derivatives of various lengths. Again, the relevance of the rather uncharacterized C-terminal part of CDKL5 is suggested by the fact that many pathogenic alterations involve the C-terminus. Importantly, in a recent publication, Bahi-Buisson and colleagues [12] analyzed patients' explants for their expression levels of CDKL5 transcripts. Some nonsense and frame shift mutations caused a significant reduction or the complete absence of the mutated transcripts, whereas other mutations, probably not subjected to the process of nonsense-mediated decay, were expressed similarly to the wild-type *CDKL5 *mRNA. Even though, so far, no reports have demonstrated the existence of the truncated CDKL5 proteins in human cells, the molecular effects of some pathogenic CDKL5 mutations (missense and truncating derivatives in the background of the CDKL5_115_ isoform) have been addressed through the overexpression of mutated derivatives in nonneuronal cell lines [33, 39–41]. To summarize such data (including our unpublished results), we can state that, as expected, missense mutations generally impair the kinase activity of CDKL5 and can therefore in most cases be considered loss-of-function mutations. Further studies are needed to evaluate whether, as hypothesized, a significant reduction of the catalytic activity of CDKL5 influences its subcellular distribution. A regulatory role for the tail of CDKL5 has emerged from the characterization of few C-terminal truncating derivatives. In fact, it seems to act as a negative regulator of the catalytic activity of CDKL5 and to modulate the subcellular localization [39, 41]. In particular, since both the pathogenic derivatives L879X and R781X confine CDKL5 to the cell nucleus, we can possibly state that the very last portion of CDKL5 acts localizing the protein to the cytoplasm. Moreover, since the leucine-rich NES-like motif, shown in Figure 2, is preserved in the L879X derivative, such motif seems insufficient for driving CDKL5 to the cytoplasm. Whether truncated CDKL5 mutants act as loss- or gain-of-function proteins still remains to be understood. Indeed, if expressed, they would be mislocalized hyperfunctional derivatives; however, as described below, since CDKL5 seems to exert its functions both in the nucleus and the cytoplasm, it remains possible that the absence of CDKL5 from the cytoplasm might also contribute to the pathogenic phenotype. To conclude and by integrating the described results with a number of studies reporting pathogenic duplications of X chromosome regions including CDKL5 [9, 42–45], it remains possible that a tight regulation of CDKL5 levels and/or activity is essential for the proper function of the central nervous system, therefore making the search for pathogenic *CDKL5 *gene duplications relevant.

So far, no clear genotype-phenotype correlation of CDKL5 mutations has been established. Some reports suggested that mutations in the COOH-terminus originate milder clinical pictures than those caused by mutations in the catalytic domain, but others state that the nature of the mutation does not correlate with the clinical heterogeneity. Accordingly, Weaving et al. [5] reported of two genetically identical CDKL5-mutated twin girls with a significant discordant phenotype. Indeed, one proband showed a clinical phenotype overlapping RTT, whereas her twin sister showed autistic disorder and mild-to-moderate intellectual disability. Since both girls were characterized by random X-inactivation, we believe that their phenotypic differences can be attributed to modifier genes that have been differentially influenced by environmental and/or epigenetic factors. Moreover, a recent report [6] identified an R952X mutation with incomplete penetrance and uncertain pathogenicity. Since this novel mutation occurs in exon 20 that, as described, might not be highly expressed in brain, we speculate that modifier genes affecting CDKL5 splicing might be responsible for the observed penetrance.

Future studies, focused on the identification of direct and indirect partners of CDKL5, will help defining its functions and might lead to the identification of modifier genes representing relevant targets for therapeutic approaches.

## 5. Modulation of CDKL5 Abundance and Localization 

Expression studies in human and mouse tissues have shown that *CDKL5/Cdkl5* mRNA is present in a wide range of tissues besides the brain, where the transcripts levels are the highest [1, 3]. Indeed, whereas *CDKL5* mRNAs can easily be detected in tissues such as testis, lung, spleen, prostate, uterus, and placenta, they are barely present, or under detection levels, in heart, kidney, liver, and skeletal muscle. Even if the data regarding CDKL5 protein expression in different tissues are somewhat conflicting, the transcription data have been partially confirmed by western blotting using adult rat extracts [24, 40]. To draw a conclusion, what is clear and commonly accepted is that, in the brain, CDKL5 levels reach the highest in conjunction with the development and differentiation of this organ. CDKL5 in fact is only weakly present during embryogenesis to get strongly induced in the early postnatal stages until P14, where after it declines [41]. 

**Figure 3:**
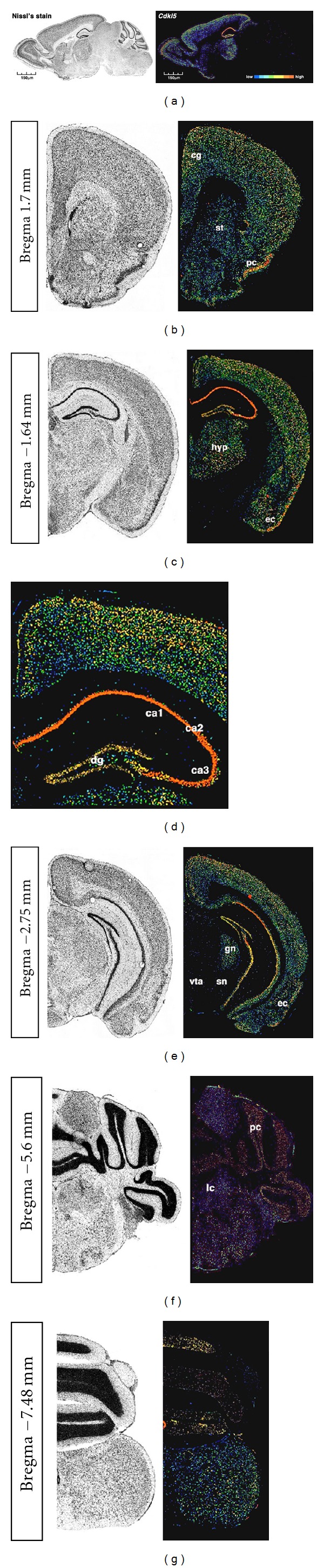
*Cdkl5 *expression patterning in the adult male mouse brain (C57BL/6J, postnatal day 56). Images are collected from the Allen Brain Atlas website (http://www.brain-map.org/; probe name RP_051219_02_C01; experiment ID 74000486 for sagittal samples, experiment ID 75042239 for coronal samples). Each Nissl-stained section is coupled by a quantitative *in situ* hybridization screening at different sectioning levels. The color bar in (a) might be used to follow transcription level intensity. (b), (c), (d), (e), (f), and (g) are representative of different brain levels, therefore, different brain areas; in particular: cg: cingulated cortex, st: striatum, pc: piriform cortex, hyp: hypothalamus, ec: entorhinal cortex, dg: dentate gyrus, ca1,2,3: hippocampal CA fields, vta: ventral tegmental area, gn: geniculate nuclei, sn: substantia nigra, lc: locus ceruleus, pc: Purkinje cells.

A detailed analysis of *Cdkl5* expression in adult mouse brain (Figure 3) shows that its mRNA levels are particularly high in the adult forebrain. Interestingly, higher expression levels are detected in the most superficial cortical layers, particularly involved in the connection of the two hemispheres through the corpus callosum; a slightly higher abundance of mRNA in the frontal cortical areas might suggest a role for CDKL5 in the physiology of such brain districts. Notably, fairly strong staining is detected in the motor cortex and the cingulate cortex, an area of high interest for the origin of a wide plethora of mental diseases. Of interest, high levels of expression are detected in the pyriform cortex and, possibly, in the entorhinal cortex. The hippocampus, a brain area that partly shares the same developmental origin as the cortex, shows very high levels of *Cdkl5 *mRNA in all the CA fields, but in the dentate gyrus, possibly in accordance with the establishment of *Cdkl5* transcription in fully mature neuronal phenotypes, given that the DG neuronal population undergoes adulthood neurogenesis. Considering the fair expression levels of *Cdkl5* in the striatum, we assume that the glutamatergic and the gabaergic neurons are by far the two cellular types expressing most of the brain *Cdkl5*. Accordingly, very low, if any, expression was detected in dopaminergic areas such as the substantia nigra or the ventral tegmental area or in noradrenergic areas such as the locus coeruleus. Of interest, however, very high levels of *Cdkl5 *transcripts are detected in several thalamic nuclei, including the geniculate nuclei. In the cerebellum, *Cdkl5* mRNA is expressed in all the lobules and, possibly, in the Purkinje cells; its levels, however, appear significantly lower when compared to the other brain areas. Specific experiments aimed at elucidating *Cdkl5 *expression in all the cerebellar cell types will shed light on its transcriptional pattern. 

Carouge and colleagues have initiated the characterization of *Cdkl5 *transcriptional regulation [46]. Interestingly, in the rat gene, they found a CpG-rich sequence of 0.8 kb (from −346 to +490), well conserved in the mouse and human counterparts. The authors demonstrated that DNA methylation involving this area inhibits *Cdkl5* expression and that the kinase gene is a target of the repression mediated by MeCP2. Even though these data contrast earlier reports where *CDKL5/Cdkl5* mRNA levels were found unaltered in RTT patient lymphocytes or brains of *Mecp2*-null mice [5, 22], we would like to mention that our unpublished results appear to confirm a role of MeCP2 in inhibiting CDKL5 expression. In the future, it might be interesting to discern whether these contradictory results stem from the capability of MeCP2 to act on different genes according to the specific cellular type. Eventually, CDKL5 transcription appears regulated upon different stimuli and depending on the specific brain district. Indeed, a significant reduction of *Cdkl5* mRNA has been observed in the striatum of rats treated with cocaine, whereas this reduction was not revealed in frontal cortexes of the same animals. As complementary results, the levels of the *Cdkl5 *transcript appear reduced incubating PC12 cells with serotonin [46].

Concerning the protein expression, the available data suggest that the levels of the kinase more or less coincide with those of mRNA in the adult brain. At the cellular level, CDKL5 is easily detectable in virtually all NeuN-positive neurons while it is expressed at very low levels in the glia [24]. 

Several mechanisms seem to regulate CDKL5 functions and levels. The presence of different CDKL5 splice variants, of which some, as mentioned, appear to be enriched in brain, indicates that alternative splicing is involved in regulating CDKL5 functions. At the functional level, we still need to understand whether the different CDKL5 isoforms have distinct functions. The only difference that has been observed so far is that CDKL5_107_ appears to be more stable than the longer human CDKL5_115_ isoform [3], whereas the subcellular localization of exogenous CDKL5 derivatives (CDKL5_115_, CDKL5_107_, and CDKL5_115+16b_) is grossly identical [3, 21].

CDKL5 functions seem to be regulated both through its subcellular localization and through its synthesis and degradation. In brain, CDKL5 is initially predominantly cytoplasmic and progressively accumulates in the nucleus, starting from roughly P14 when approximately 40% of total CDKL5 can be detected in this compartment. However, CDKL5 gets significantly translocated to the nucleus only in certain brain areas: in the cerebellum, for example, more than 80% of CDKL5 remains cytoplasmic while in the cortex it is almost equally distributed between cytoplasm and nucleus [41]. Exogenous CDKL5 shuttles constitutively between the two main compartments in cultured nonneuronal cells through an active nuclear export-dependent mechanism involving the C-terminus of the protein and the CRM1 nuclear export receptor. Interestingly, however, in resting hippocampal neurons the protein is not dynamically shuttling and its nuclear exit appears to be regulated by specific stimuli. In fact, glutamate treatment induces nuclear export of CDKL5 leading to its accumulation in the cytoplasm [47]. In the future, it will be interesting to understand whether (a) posttranslational modifications or interactions with other proteins are involved in regulating the nuclear export/import of CDKL5, (b) the enzymatic activity of the protein is modulated by its localization and interaction with other factors, and eventually (c) which other stimuli influence the localization and activity of CDKL5.

As mentioned, the degradation of CDKL5 seems also to be involved in regulating its functions. The long CDKL5_115_ isoform is, in fact, rapidly degraded in a proteasome-dependent manner in transfected cells. Conversely, the human and mouse CDKL5_107_ isoforms are more stable and their quantity is not significantly increased by the proteasome inhibitor MG132, thus indicating that the very C-terminal region, from amino acid 905, contains signals responsible for this degradation [3]. In neurons, however, CDKL5 levels are strongly reduced upon sustained glutamate treatment or other cell death signals [47]. Why neurons degrade CDKL5 upon cell death induction and how this degradation is regulated still remains to be elucidated.

## 6. Is CDKL5 Involved in Synaptic Function, Structure, and Plasticity? 

Modulation of cellular activity is the force that regulates dendritic growth and morphology during neuronal development, affecting locally the synapses and regulating transcription at the nuclear level. Similarly, activity-induced calcium influx alters synaptic function in mature neurons, modifying synaptic strength and gene expression [48]. Therefore, extracellular cues and neuronal adaptive responses equally cooperate through signaling mechanisms that flow from the synapses through the cytoplasm to, eventually, the nucleus. Mutations in components of these signaling pathways might thus result in impaired information processing in the brain, therefore leading to neurodevelopmental, neuropsychiatric, and neurodegenerative disorders. In particular, Rett syndrome, whose connection with CDKL5 has already been discussed, is characterized by a number of synaptic deficits [49]. Is CDKL5 actually involved in neuronal plasticity? Even though the lack of mouse models for *Cdkl5 *functions does not permit to draw a definitive picture, there are several pieces of evidence supporting this hypothesis.

### 6.1. Cdkl5 Expression Correlates with Neuronal Maturation and Maintenance

In recent years, it has become clear that dendrites and spines are dynamic structures that during early postnatal development undergo a significant remodeling necessary for synapse function and plasticity. As development proceeds up to the adulthood, spines continue to be remodeled in response to diverse stimuli such as LTP and LTD; these changes are considered of high relevance for learning and memory.

As already mentioned, CDKL5 expression correlates, both *in vitro *and *in vivo,* with neuronal maturation, reaching the highest levels of expression when neurons acquire a mature phenotype and suggesting an involvement of the kinase in neuronal differentiation and arborization [24, 41, 47]. Interestingly, even if CDKL5 levels seem to slightly decrease in adult brain, they are significantly higher when compared to nonneuronal tissues. Therefore, it is possible to speculate that CDKL5 might play a role in maintaining neuronal functions in addition to maturation. Furthermore, since, as already stated, CDKL5 intracellular distribution changes upon neuronal maturation and its nuclear fraction peaks in adult brain, such fraction may be involved in adult synaptic plasticity.

### 6.2. Cdkl5 Affects Neuronal Morphogenesis through Cytoskeleton Rearrangements

The morphology of neuronal extensions and their spines influences the electrophysiology and behavioral output of the cell. Indeed, arborization defects have already been reported in brain disorders, such as RTT and Fragile X, in which experience-dependent neuronal maturation and plasticity are disrupted [50]. Interestingly, by RNAi and overexpression of CDKL5 in cultured rat cortical neurons, Chen and colleagues demonstrated that CDKL5 is a critical regulator of neuronal morphogenesis and dendritic arborization. *In vivo*, the kinase shows overlapping functions and affects neuronal migration [24]. Even if future studies should investigate whether a malfunction of CDKL5 also impairs synaptic spine morphology, it is important to mention that CDKL5 colocalizes with F-actin in the growth cone and interacts with Rac1 [24]. Rac1 belongs to the Rho GTPase family of proteins that promotes the formation and/or maturation of spines by remodeling the actin cytoskeleton of neuronal spines [51]. Functional experiments suggested that CDKL5 influences neuronal morphogenesis by acting upstream of Rac1.

### 6.3. CDKL5 Functions Respond to Neuronal Stimuli

A comprehensive knowledge of the stimuli affecting CDKL5 expression and activities will help to understand how its deficiency impacts brain functions. However, so far, only limited information is available. In particular, it has been demonstrated that *BDNF*, an activity-regulated gene encoding a neurotrophin already involved in several neurological and psychiatric disorders including RTT, induces a transient phosphorylation of CDKL5. The kinase is required for the capability of BDNF to activate Rac1 [24]. Furthermore, we demonstrated that CDKL5 is transported outside the nucleus into the cytoplasm in response to activation of extrasynaptic NMDA receptors (NMDA-R). Recent publications suggest that extrasynaptic NMDA-Rs have a role in LTD and dephosphorylation of CREB; alterations in the cross-talk between synaptic and extrasynaptic receptor activities might play an important role in seizures [52–54]. Of interest, we discovered that CDKL5 is degraded by the proteasome in response to extended glutamate bath stimulation or other death stimuli [47]. These results, linking CDKL5 to programmed cell death pathways, appear particularly intriguing considering that (1) local cell death and pruning enable proper brain development and (2) proteolysis by the ubiquitin-proteasome pathway is emerging as a new mechanism controlling synaptic plasticity. As an example, the ubiquitin ligase Ube3A is associated with human cognitive defects, including Angelman syndrome, and it has been hypothesized that defective degradation of different Ube3A substrates, such as Arc, might contribute to the Angelman clinical manifestations [55].

### 6.4. CDKL5 Might Regulate the Functions of Epigenetic Factors and Transcriptional Regulators

As already mentioned, bidirectional signaling pathways link synapses to the nucleus, and, moreover, sensory experiences influence gene expression. Regarding this topic, little evidence suggests that CDKL5 has a role in regulating gene expression. Chen and colleagues [24] demonstrated that the ability of CDKL5 to influence dendritic growth occurs both in the cytoplasm and in the nucleus and requires its catalytic activity. Accordingly, we, and others, have demonstrated that, in cultured cell lines, exogenously expressed CDKL5 interacts with the transcriptional repressor MeCP2 [22, 40] and seems capable of phosphorylating the methyl binding protein *in vitro*. Indeed, the two human CDKL5 isoforms (CDKL5_107_ and CDKL5_115_) immunopurified from cultured cell lines induce the phosphorylation of a recombinant MeCP2, purified from *E. coli *[3, 22, 39]. Identical results are routinely obtained in our lab using the murine CDKL5 (unpublished results). To this regard, two previous publications were unable to obtain a CDKL5-mediated phosphorylation of MeCP2 [40, 56]. It is, however, important to mention that the experimental conditions were quite different; indeed, one paper was using a truncated CDKL5 derivative that lacks the identified MeCP2-interacting domain [56], the second one uses a lower abundant, immunopurified MeCP2 [40]. It is still unclear whether CDKL5 influences the phosphorylation state of MeCP2 *in vivo*. Anyhow, it must be recalled that several papers suggest that, in neurons, MeCP2 functions as a dynamic epigenetic factor, regulating gene transcription during learning and memory through activity-dependent phosphorylation of specific serine residues [57–60]. The neuronal activity-induced phosphorylation of MeCP2 is required for proper dendritic/synaptic development and behavioral responses to experience [61, 62]. Intriguingly, recent evidence suggests that postmitotic neurons exploit epigenetic mechanisms, including DNA methylation, to consolidate and stabilize cognitive-behavioral memories. Indeed, inhibition of DNA methyltransferases (DNMT) in the hippocampus affects contextual fear memories and LTP [63]. The connection of CDKL5 to epigenetics and gene expression seems also reinforced by its possible interaction with DNMT1 [56] and by the already mentioned induction of *Cdkl5 *expression in response to MeCP2 ablation, DNMT inhibition, and histone deacetylase inhibition [46]. 

**Figure 4:**
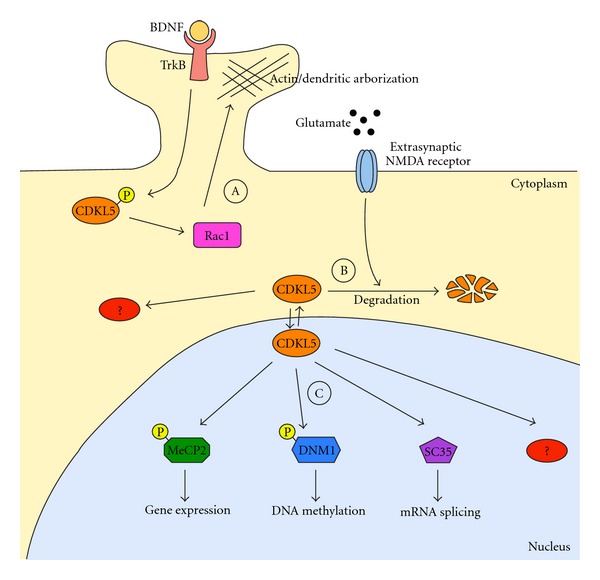
A model depicting the different functions of CDKL5 in the cytoplasmic and nuclear compartments. As described in the text, CDKL5 functions are occurring both in the cytoplasm and in the nucleus. A. In the cytoplasm, CDKL5 is involved in the regulation of actin cytoskeleton and dendritic arborization. This function is mediated by the interaction of CDKL5 with Rac1. Importantly, a link between BDNF, CDKL5 phosphorylation, and Rac1 activation has been suggested by Chen et al. [24]. B. In the cytoplasmic compartment, the levels of CDKL5 are regulated by degradation. Furthermore, we assume that several phosphorylation targets of CDKL5 remain to be identified. C. In the nucleus, CDKL5 has been proposed capable of interacting with and phosphorylating MeCP2 and DNMT1, thereby influencing gene expression and DNA methylation. Furthermore, the protein has been shown to colocalize with RNA speckles involved in RNA splicing. As for the cytosol, we assume that several other targets of CDKL5 remain to be discovered. In this model, we have neglected the capability of the nuclear CDKL5 to influence dendritic arborization [24] and the transcriptional regulation of *CDKL5*.

To conclude, we believe that CDKL5-signaling cascades are involved in synaptic plasticity and learning, affecting spines, dendritic branching, and actin cytoskeleton in the cytoplasm and activity-dependent gene expression in the nucleus (Figure 4). Clinical, genetic, and biological data suggest a functional relationship between CDKL5 and MeCP2, leading to hypothesize that common biological network(s) are disrupted when either gene is mutated. We speculate a possible mechanism in which CDKL5 works upstream of MeCP2, directly or indirectly influencing its phosphorylation state and activity. According to this model, while mutations in *MECP2 *mainly lead to classic RTT, altered CDKL5 activity would determine, among others, certain phosphorylation-dependent MeCP2 dysfunctions, bringing to a subset of RTT symptoms. Other uncharacterized CDKL5 targets could also be deregulated leading to phenotype specifically associated with mutations in CDKL5. However, as already proposed [46], a transcriptional control of MeCP2 over *CDKL5 *might also exist, therefore, reinforcing the link between the two proteins. The two models should not be considered mutually or temporally exclusive. Considering all above, the identification of the cytoplasmic and nuclear phosphorylation targets of CDKL5, together with possible transcription events directly or indirectly affected by the kinase, represents an important future milestone towards understanding its neuronal functions.
